# Histomorphological effects of the oil extract of *Sphenocentrum jollyanum* seed on benign prostatic hyperplasia induced by exogenous testosterone and estradiol in adult Wistar rats

**Published:** 2019

**Authors:** Godwin Mbaka, Steve Ogbonnia, Adeola Sulaiman, Daniel Osiagwu

**Affiliations:** 1 *Department of Anatomy, Lagos State University College of Medicine, Ikeja, Lagos, Nigeria*; 2 *Department of Pharmacognosy, University of Lagos, Idi-Araba, Lagos, Nigeria*; 3 *Department of Anatomy, Olabisi Onabanjo University, Remo Campus, Ogun State, Nigeria*; 4 *Department of Anatomic and Molecular Pathology, College of Medicine of the University of Lagos, Nigeria*

**Keywords:** Histomorphology, Sphenocentrumjollyanum seed, Phytotherapy, Benign prostatic hyperplasia, Male rats

## Abstract

**Objective:**

*Sphenocentrum jollyanum* (SJ) seed has many health benefits due to its very potent anti-inflammatory and antioxidant properties. Despite its widespread use, it has not been validated for use in the treatment of benign prostatic hyperplasia (BPH). This study was conducted to examine histomorphological effects of SJ seed on BPH that usually causes bladder outlet obstruction.

**Materials and Methods:**

There were a total of six groups of animals each comprising 5 adult male rats. Apart from group 1 (normal control), in the remaining five groups, BPH was induced. Group 2 (negative control) was sacrificed immediately after BPH induction; groups 3 and 4 received the extract at 300 and 600 mg/kg respectively by gavages for thirty days; group 5 received finasteride (0.1 mg/kg) for thirty days and group 6 received the extract (600 mg/kg) simultaneously with the steroid administration for thirty days. The animals’ were weighed before the experiment and subsequently every three days until the end of the study.

**Results:**

The extract caused marked decrease in prostate weight of rats with BPH with histo-morphology of the tissue showing degenerated stromal and epithelial cells with few epithelial involutions of glandular tissue. Prostate specific antigen (PSA) level as well as testosterone level significantly (p<0.05) decreased in the treated groups compared to negative control. BPH animals treated with extract/finasteride exhibited remarkable increases in anti-oxidant enzymes level with concurrent decreases in peroxidative activity.

**Conclusion:**

SJ effectively ameliorated prostatic hyperplasia in BPH animals causing marked degenerative changes in prostate stromal and epithelial cells and also exhibited marked anti-oxidant effect.

## Introduction

Benign prostatic hyperplasia (BPH) is a noncancerous cell growth in the prostate gland. This clinical condition elicited by uncontrolled growth of glandular epithelial and stromal cells, induces lower urinary tract symptoms (LUTS) which include weak urinary stream, incomplete bladder emptying, nocturia, dysuria and bladder outlet obstruction (Pais, 2010 ▶; Roehrborn, 2011 ▶). These symptoms are worrisome conditions that can cause significant impact on the quality of life. Research on BPH was initiated on the realization that enlargement of this organ could result in obstruction of bladder outlet (Lawson, 1986). Since then, numerous anatomical, pathological, and clinical studies have been conducted to investigate BPH and its clinical manifestations in patients. Despite various research endeavors, the etiology of BPH is yet to be fully understood. The constraint is partly linked to the organ that it affects (i.e. the penis) and the symptoms it presents which makes most men find it difficult to talk to their doctors about it and so, there has being no proper documentation of its history and course of development (Mbaka et al., 2013 ▶). The disease has shown to have age-related prevalence as it is the most common ailment in elderly men. It has been observed that at the age of 50 years old and above, there is an estimated prevalence of about 40 % while at the age of 80 years old and above, about 90 % of men manifest the health conditions (Bhargava et al., 2004 ▶; Roehrborn and Rosen, 2008 ▶). 

BPH development has been established to be influenced by hormonal changes in ageing men. Specifically by the stimulation of prostatic androgen dihydrotestosterone (DTH), an active metabolite formed by enzymatic conversion of testosterone by steroid 5α-reductase enzyme (Russel and Wilson, 1994 ▶). Following increased formation and accumulation of DTH in the prostate that shows an age dependent increase, it may trigger stromal and glandular cells proliferation and possibly cause nodular hyperplasia in the affected men (Carson and Rittmaster, 2003 ▶). 

Management options for prostatic hyperplasia include surgical intervention and orthodox drug treatment. Therapeutic agents available for treatment of BPH include 5α-reductase inhibitors that appear very efficient because they inhibit the development of BPH via a reduction in DHT production (Gravas and Olke, 2010 ▶). Despite the efficacy of orthodox medication, they have shown increased risk of systemic side effect like dizziness, erectile dysfunction, decreased libido, etc. (Naslund et al., 2007 ▶). These shortcomings made the search for alternative therapies more compelling. The use of alternative therapy in BPH management is an ancient practice. In most developing economies, particularly in Africa, alternative therapy is the predominant mode of managing BPH because of the public belief in the efficacy of herbal therapeutic agents (Odugbemi, 2006 ▶). 

*Sphenocentrum jollyanum* (SJ) Pierre (Menispermaceae) is a perennial plant that grows naturally along the West Coast sub-region of Africa that includes Nigeria. It is deep rooted with few branches bearing fruits that contain a single large oval shaped seed (Mbaka and Owolabi, 2011 ▶). It is used for dressing wounds particularly chronic wounds, and against feverish conditions, and cough; also it is consumed as an aphrodisiac agent (Dalziel, 1955 ▶; Iwu, 1993 ▶). Studies have shown the seed to possess significant antipyretic and analgesic activities (Dalziel, 1955 ▶; Muko et al., 1998 ▶). The seeds also showed significant antioxidant (Nia et al., 2004) and anti-inflammatory (Moody, 2006 ▶) properties which led to isolation of very potent anti-inflammatory furanoditerpenes identified as columbin, isocolumbin and fibleucin (Moody, 2006 ▶). Considering several health benefits of SJ seed, its very potent anti-inflammatory and antioxidant properties, we investigated its efficacy in managing BPH. To the best of our knowledge, no evidence is available in this regard.

## Materials and Methods

**Plant materials**

The dried seeds of SJ were purchased from Ibode market in Ibadan, Oyo State, Nigeria. They were authenticated by a taxonomist at the Forestry Research Institute of Nigeria (FRIN), Ibadan where a voucher specimen was deposited (FHI/108203).

**Preparation of the petroleum ether extract of** ***Sphenocentrum*** ***jollyanum*** **seed**

A smooth powder was made of the dried seeds using an electric grinder. The seed powder, (1.57 kg) was extracted by petroleum ether (at 60-80 ^o^C) in three cycles using a Soxhlet extractor. The crude oil extract of SJ seed was concentrated *in vacuo* at 30 ^o^C to obtain 146 g residue which was an equivalent of 9.3 % w/w. The residue was stored in an air-tight bottle kept in a refrigerator at 4 ^o^C till used.

**Animals**

Adult male rats (200 - 210 g) obtained from the Animal House of the University of Ibadan, Oyo State, Nigeria, were kept under standard environmental conditions of 12 hr /12 hr light/dark cycles. They were housed in polypropylene cages (5 animals per cage), and maintained on mouse chow (Livestock Feeds Nigeria Ltd) and provided with water *ad libitum*. They were allowed to acclimatize for 10 days to the laboratory conditions before initiation of the experiment. The use and care of the animals, and the experimental protocols were in compliance with the Institute of Laboratory Animals Research (ILAR) guidelines on the use and care of animals, in experimental studies (ILAR, 1996 ▶).

**Testosterone**

This is a steroid hormone from the androgen group and is found in mammals, reptiles and other vertebrates (Dabbs, 2000 ▶). In mammals, testosterone is primarily secreted in the testes of males and the ovaries of females, although small amount is also secreted by the adrenal glands. It is principally male sex hormone and anabolic steroid which play vital role in the development of male reproductive tissues such as testes and prostate (Nelson, 2005 ▶). 

**Estradiol**

It is a steroid hormone often called “female” hormone but also present in the males representing the major estrogen in humans (Guyton and Hall, 2000 ▶). There are some evidences that estrogen may play a role in the etiology of BPH. This effect appears to be mediated mainly through local conversion of estrogen to androgens in the prostate tissue rather than a direct effect of estrogen itself (PentikÃ¤inen et al., 2006 ▶).

**BPH Induction**

BPH was induced by administration of exogenous testosterone, 0.32mls from the stock containing 300 µg of testosterone and estradiol, 0.2mls from the stock containing 60 µg of estradiol at staggered doses (three times a week) for three weeks (Mbaka et al., 2017 ▶). The steroid hormones were diluted with corn oil used as the solvent. 

**Animal grouping and treatment**

There were a total of six groups, each comprised of 5 adult male rats. Apart from group 1, the normal control (intact), in the remaining five groups, BPH was induced. Group 2 (negative control) was sacrificed immediately after BPH induction while groups 3 and 4 received the extract at 300 and 600 mg/kg body weight (bwt) doses, respectively by gavage for thirty days; group 5 received finasteride (0.1 mg/kg) for thirty days and group 6 received the extract (600 mg/kg) with simultaneous steroids injection (BPH induction) for thirty days. The animals were weighed prior to the commencement of the experiment and subsequently every three days till the end of the experiment. 

**Assay for testosterone and prostate specific antigen (PSA)**

Enzyme immunoassay technique was used for the quantitative determination of testosterone concentration and PSA evaluation (Marcus and Dumford, 1985 ▶; Ekins, 1990 ▶). 

**Oxidative activities**

The oxidative activity was assessed after overnight fasting. The animals were sacrificed and the harvested hepatic tissue were homogenized and used for the assays.

*Superoxide dismutase assay*

Superoxide dismutase (SOD) was assayed utilizing the technique of Kakkar (Kakkar et al., 1984 ▶). A single unit of the enzyme was expressed as 50 % inhibition of Nitroblue Tetrazolium (NBT) reduction/min/mg protein which was measured spectrophotometrically at 420 nm.

*Catalase assay*

Catalase (CAT) activity was assayed colorimetrically at 620 nm and expressed as μmoles of H_2_O_2_ consumed/min/mg protein (Rukkumani et al., 2004 ▶). The hepatic tissue was homogenized in isotonic buffer (pH 7.4). The homogenate was centrifuged at 1000 rpm for 10 min. The reaction mixture contained 1.0 ml of 0.01M phosphate buffer (pH 7.0) which was added 0.1mlof tissue homogenate and 0.4 ml of 2.0 ml of dichromate-acetic acid reagent (5 % potassium dichromate and glacial acetic acid mixed at 1:3 ratio).

*Estimation of glutathione*

The glutathione (GSH) level was determined by the method of Ellman (Rukkumani et al., 2004 ▶). To the hepatic homogenate 10 % trichloroacetic acid (TCA) was added and the mixture was centrifuged. Next, 10ml of the supernatant was treated with 0.5 ml of Ellmans’ reagent in 100 ml of 0.1 % of sodium nitrate and 3.0 ml of phosphate buffer (0.2M, pH 8.0). The absorbance was read at 412 nm.

*Estimation of lipid peroxidation*

Lipid peroxidation as evidenced by the formation of thiobarbituric acid reactive substances (TBARS) and hydroperoxides (HP) were measured by the method of Niehaus and Samuelsson (Rukkumani et al., 2004 ▶) and expressed as nmol/ml. In brief, 0.1 mL of hepatic tissue homogenate (Tris-Hcl buffer, pH 7.5) was treated with 2 ml of (at 1:1:1 ratio) TBA-TCA-HCl reagent (containing thiobarbituric acid 0.3 %, 0.25N HCl and 15 % TCA) the homogenate was centrifuged at 1000 rpm for 10 min. The absorbance of clear supernatant was measured against reference blank, at 535 nm. 

**Prostate tissue preparation for histological examinations**

The prostatic tissue harvested from each animal group was fixed in 10 % formal saline solution for seven days. Before embedding in paraffin wax, the fixed prostate tissue was removed and dehydrated in increasing concentrations of ethanol; (70 %, 80 %, 90 % and 100 %). The tissue was thereafter treated with acetone and then cleared in xylene for 30 min to enhance the tissue transparency followed by impregnation and embedding in paraffin wax. The embedded tissue was sectioned at 5 μm, mounted on a slide and stained with hematoxylin and eosin (H&E) stains (Mbaka et al., 2017 ▶). Each section was examined under a light microscope for structural changes and photomicrographs were taken. 

**Statistical analysis**

All values were expressed as mean±standard error of mean and the statistical significance between treated and control groups were analyzed by means of Student’s t-test. A p<0.05 was considered significant.

## Results

**The animal body and prostate weights**

**Figure 1:**
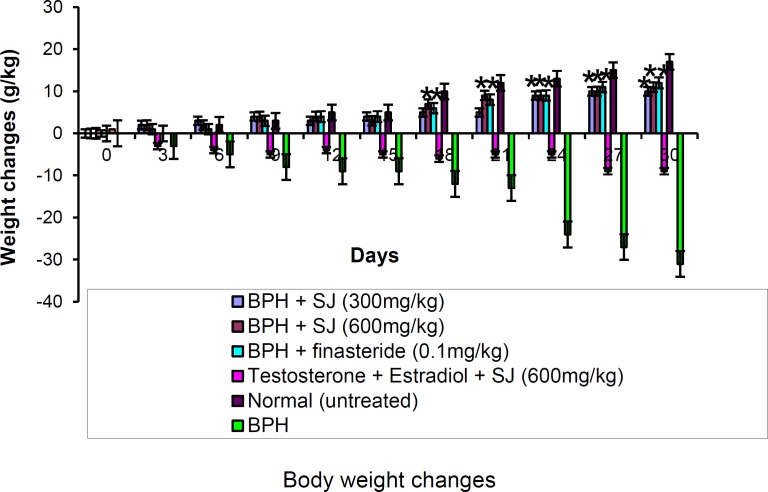
Change in body weight of animals both in control and treated. *p≤ 0.05 as compared to BPH control

The body weight changes of the control and the groups that received SJ oil/finasteride are shown in Figure. 1. 

During the three weeks of BPH induction, the quantity of food consumed by the animals decreased considerably which reflected in the animals’ body weight decrease. The BPH animals treated with SJ oil/finasteride exhibited gradual weight gain as well as improvement in appetite while the untreated BPH animals showed progressive body weight decrease. 

**Figure 2:**
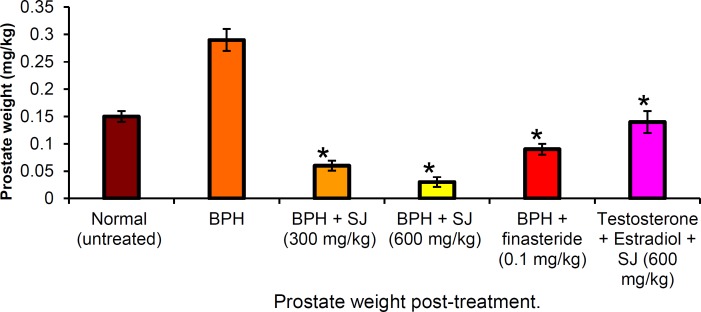
Absolute prostatic weight in the treated and untreated animals after 30 days. *p ≤ 0.05 as compared to BPH control

There were also prostatic weight changes as indicated in Figure. 2. The BPH animals showed significant (p<0.05) increase in prostate weight compared to the normal animals. The animals treated with extract/finasteride on the other hand exhibited prostatic weight decrease. The SJ oil-treated animals showed dose-dependent (300 and 600 mg/kg) prostate weight decrease of 79.3% and 89.7%, respectively compared to negative control. The finasteride-treated animals showed a decrease of 68.9% indicating that the orthodox drug exhibited comparatively less activity compared to SJ oil extract.

**Effect of SJ seed oil on PSA level**

**Figure 3:**
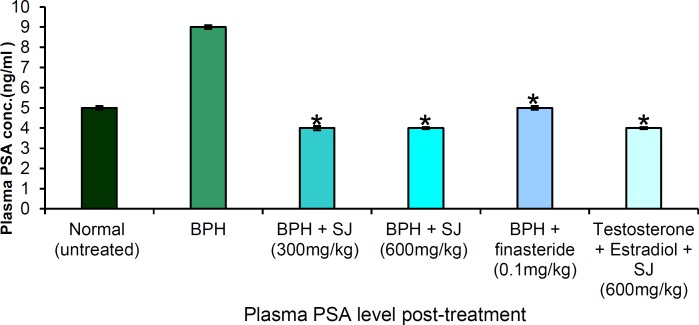
Plasma PSA level in the treated and untreated animals after 30 days. * p ≤ 0.05 as compared to BPH control

Figure 3 shows the plasma PSA levels in the treated and control groups. There was an increase in PSA level to 9.0 ng/ml after BPH induction. In the SJ oil/finasteride treated, significant (p<0.05) decreases in PSA level occurred compared to the negative control. The two extract doses (300 and 600 mgkg) exhibited PSA reduction of 55.5% (4 ng/ml) and 55.5% (4 ng/ml), respectively; finasteride-treated rats showed 44.4% (5 ng/ml), while simultaneous extract administration with BPH induction for thirty days resulted in 55.5% (5 ng/ml).

**Effect of SJ seed oil on testosterone level**

Figure, 4 shows the effect of SJ oil/finasteride on testosterone level of BPH animals. In BPH animals, the testosterone level was elevated to 5.7 ng/ml. In the extract-treated, dose-dependent decrease occurred (300 and 600 mg/kg) indicating 68.4% (1.8 ng/ml) and 70.2% (1.7 ng/ml) respectively. Finasteride treatment decreased testosterone level by 70.2% (1.7 ng/ml), extract administration with simultaneous BPH induction for thirty days showed decrease in testosterone level by 45.6% (3.1 ng/ml).

**Figure 4:**
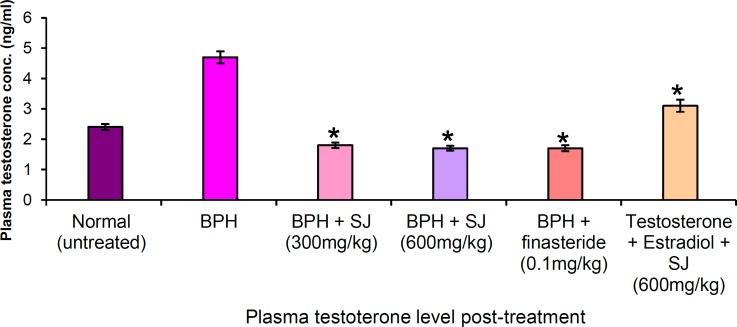
Plasma testosterone level in the treated and untreated animals after 30 days. *p ≤ 0.05 as compared to BPH control

**Effect of SJ seed oil on antioxidant profile**

**Figures 5:**
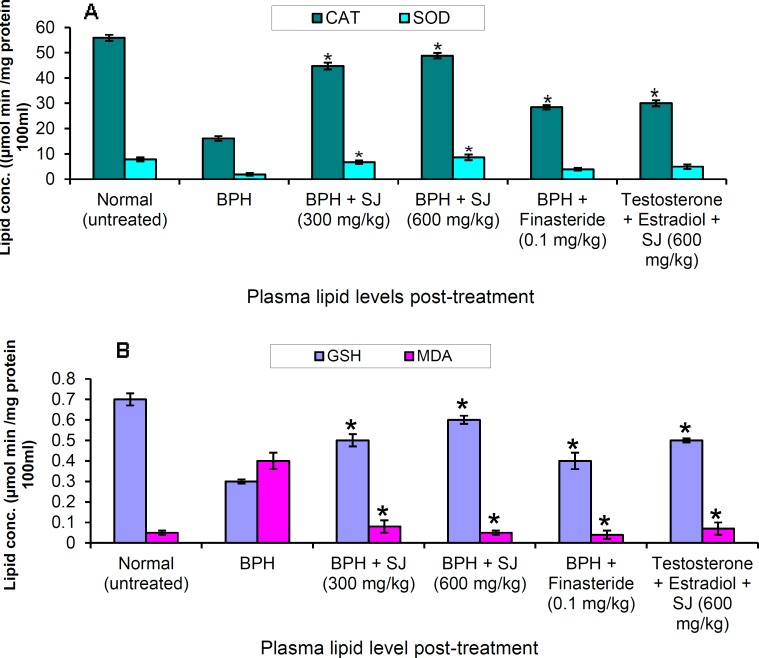
A and B. Plasma lipid levels in the treated and untreated after 30 days. *p ≤ 0.05 as compared to BPH control

Oxidative stress occurred following BPH induction which resulted to alteration in the homeostasis of antioxidant enzymes. The activity of the enzymes, CAT, SOD and GSH (Figures. 5a, and b) decreased markedly in the untreated BPH groups. However, in the group treated with SJ oil/finasteride, the activity of the enzymes was significantly (p<0.05) restored compared to the negative control. Increases in the enzymes activity was more marked following treatment with SJ oil compared to the orthodox drug. In simultaneous extract administration with BPH induction for thirty days, the level of the antioxidant enzymes was comparably lower than the normal rats. The TBARS evaluation showed increases in peroxidative activity in the untreated animals whereas in SJ oil/finasteride treated rat, peroxidative activity showed marked decreases to a level comparable to that of normal animals.

**Histology/histopathology of prostatic tissues**

**Figure. 6:**
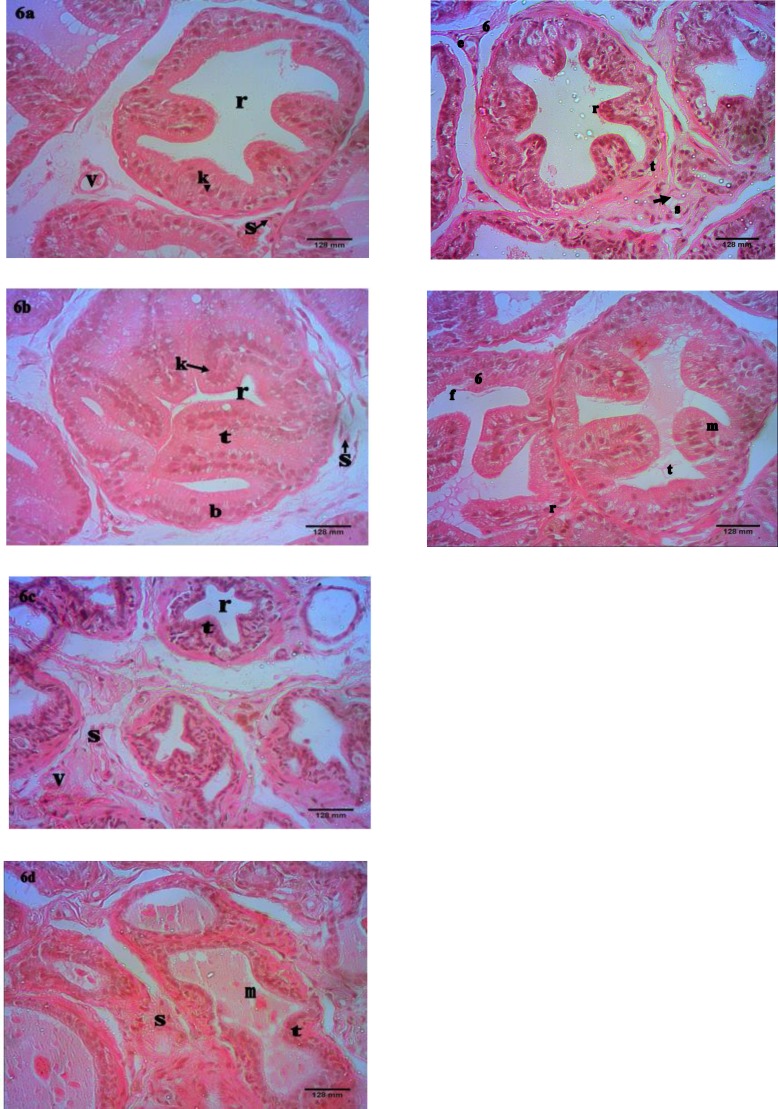
a) Photomicrograph of a cross section of normal prostate gland indicating stromal cells (s), thick intraglandular epithelia involutions highlighting epithelial cells (k), gland lumen (r) and blood vessels. (H and E stained) X400. b) A cross section of BPH gland indicating epithelial proliferation with overlapping involutions (t), partially obliterated gland lumen (r), secretory cells (k), basal cells (b) and stromal cells (s). (H and E stained) X400. c) A cross section of BPH gland treated with low extract dose indicating marked decreases in intraglandular epithelium (t) with fewer involutions and increased gland lumen (r). Blood vessel (V) and stromal cells (S) are indicated in the fibromuscular matrix. (H and E stained) X400. d) A cross section of BPH gland treated with high dose of extract showing intraglandular epithelium (t), large lumen with few involutions, corpora amylacea (m) and interglandular stromal cells (s). (H and E stained) X400. e) A cross section of finasteride-treated BPH gland showing decreased intraglandular epithelium (t), few involutions and increased intraglandular lumen (r), stromal cells (s) in fibromuscular matrix. (H and E stained) X400. f) A cross section of the group administered with the steroids and simultaneously treated with the extract indicating thick intraglandular epithelium (t), intraglandular lumen (r) and corpora amylacea (m). (H and E stained) X400

All the animals that were administered with testosterone and estradiol, exhibited prostatic hyperplasia after three weeks of the steroids administration except for the group simultaneously induced and administered with SJ oil extract which exhibited mild BPH. The normal prostat**e **tissue stained with H and E (Figure. 6a) showed deeply stained glandular epithelium that made regular involutions into the lumina. The stroma made of connective tissues fibres and smooth muscle cells, showed sizeable proportion while the fibromuscular matrix showed the presence of blood and lymphatic vessels. 

The photomicrograph of the negative control (Figure. 6b) showed serious disruption in the histo-architecture of the prostatic tissue indicating stromal and epithelial proliferations leading to glandular hyperplasia. The epithelium which was much thicker compared to normal prostate had higher numbers of columnar cells with vacuoles. It showed large and overlapping involutions that markedly reduced the volume of the lumen making it partially obliterated. The stromal cells were also highlighted in the fibro-muscular matrix.

The prostatic tissue histology of animals treated with low dose of the oil extract (Figure. 6c) showed significant improvement in the histo-architecture of the prostatic tissue with marked reduction in epithelial thickness and with fewer involutions. The stromal cells showed normal appearance while the glandular lumen showed increased volume compared to the negative control. There were also structural changes in the fibro-muscular matrix that showed increased density comparable to normal.

In rats that received the higher dose of the extract (Figure. 6d), there was degeneration of stromal and epithelial cells with the epithelial lining reduced to a thin layer with very few involutions. The glandular lumen on the other hand, exhibited significant enlargement in size. 

The prostatic tissue (Figure. 6e) of finasteride-treated rats exhibited marked reduction in stromal and epithelial cell sizes compared to the negative control but showed less activity when compared to animals treated with SJ oil extract. 

In the extract administration with simultaneous BPH induction for thirty days (Figure. 6f), partial glandular hyperplasia occurred. Luminal epithelial involutions were observed which was comparably higher than the normal animals.

## Discussion

The use of lipid extract of plant in treating BPH has attracted marked attention because of the successes recorded overtime. Lipid extracts from Saw palmetto and Cuban Royal palm have long been used as a natural herbal remedy for prostate enlargement. They have been established to contain active component that gradually and significantly reduce prostate mass and reverse the degenerative changes in the structure of the prostate gland by inhibiting 5α-reductase activity (Strauch et al., 1994 ▶; Carbajal et al., 2004 ▶). Similarly, pumpkin seed oil (*Cucurbita pepo*) has been reported to block testosterone/prazosin- induced increase in rat prostate weight (Tsai et al., 2006 ▶). The efficacy of these herbal lipid therapies drew attention to lipid products of plant sources to be used for BPH treatment. 

In this study, BPH was induced using testosterone and estradiol leading to hyperplasia of prostate gland and body weight loss. Prostate weight increase is often considered a reliable index of BPH development (Veeresh et al., 2010 ▶). The body weight loss in this case may be due to decrease in appetite during BPH induction. The characteristic of BPH is epithelial and stromal cells hyperplasia which causes the enlargement of prostate gland. In the negative control (BPH animals), the tissue histology showed extensive stromal and epithelial proliferation resulting in partial obliteration of the glandular lumen. Stromal cells activity is an important issue because they regulate epithelial growth and support and maintain epithelial function; hence, BPH is considered an intrinsic mesenchymal disease because of the inductive interactions between prostatic stroma and the epithelium (Zhao et al., 2007 ▶). On the other hand, the animals treated with SJ seed oil extract/finasteride for thirty days exhibited marked changes in histo-architecture of prostatic tissue. The treatments led to significant decreases in stromal hyperplasia with decreased epithelial thickness. There was also reduction in epithelial involutions with consequent increases in the volume of glands lumen. The changes in histo-architecture of the extract-treated rats probably reflected the inhibition of 5α-reductase activity. A wide range of herbal therapies reported for use in BPH management modulate hyperplasia by inhibiting 5α-reductase activity (Strauch et al., 1994 ▶; Veeresh et al., 2010 ▶; Nandecha et al., 2010 ▶). Finasteride has also been reported to modulate hyperplasia by similar mode of activity (Strauch et al., 1994 ▶; Afriyie et al., 2014 ▶). It is understood that prostate gland contains 5α-reductase enzymes that converts testosterone to DHT, a potent androgen that plays a critical role in the pathogenesis of prostatic stromal and epithelial cell growth which consequently results in BPH (Carson and Rittmaster 2003 ▶). SJ seed oil equally exhibited considerable effective prophylaxis because it did inhibit BPH progression in simultaneous induction with the extract treatment.

There was an increase in PSA level in the BPH animals but after thirty days of treatment with SJ oil/finasteride, marked decreases occurred. PSA is a glycoprotein produced in low quantities by cells of the prostate gland and is present in serum which could be used as a semi-quantitative indicator or surrogate marker for prostatic volume in BPH and prostatic cancer (McPartland and Pruitt, 2000 ▶). It is also considered as a predictor of increased risk of acute urinary retention (Roehrborn, 2001 ▶). Although there might be a good correlation between prostate volume and serum PSA level (Öesterling et al., 1993 ▶), the use of PSA level alone as an indicator for prostate enlargement might not be sufficient. It is because much remains unknown about the interpretation of PSA levels as it pertains to test’s ability to discriminate cancer from benign prostate conditions, and the best course of action following finding elevated PSA levels. 

The availability of free (active) testosterone in the blood stream is noted for its key role in BPH progression. Studies showed that high levels of free testosterone promote the stimulation of 5α-reductase found mainly within the stromal cells which converts testosterone to a potent androgen, DHT that is responsible for the pathogenesis of prostate gland (Wong et al., 2000 ▶). In the animals treated with SJ oil/finasteride, there was a considerable decrease in testosterone level. The reason for the decrease was not clear. However, it might be attributed to SJ oil/finasteride activity which may have mopped up circulating testosterone that could constitute a risk factor for hyperplasia of prostate gland. 

Several studies have shown that BPH is associated with increased oxidative stress which increases with increasing age (Aydin et al., 2006 ▶; Aryal, 2007 ▶). In BPH, increased lipid peroxidation and decreased levels of superoxide dismutase and antioxidant molecules, have been documented (Aydin et al., 2006 ▶; Aryal, 2007 ▶; Almushatat et al., 2006 ▶). The BPH animals showed marked decreases in the levels of anti-oxidant enzymes, CAT, SOD and GSH which apparently could be due to accumulation of superoxide anion radicals and hydrogen peroxide that accentuate peroxidative activity (Halliwell and Gutteridge, 1985 ▶). The BPH animals that received SJ oil/finasteride treatments exhibited remarkable increases in the anti-oxidant enzymes level with concurrent decreases in peroxidative activity. SJ seed extract is recognized for its very potent anti-inflammatory and antioxidant activities and it has been shown to be rich in furanoditerpenes identified as columbin, isocolumbin and fibleucin (Nia et al., 2004 ▶; Moody, 2006 ▶). Plants rich in anti-inflammatory and antioxidant compounds have shown to be useful agents in ameliorating oxidative stress in BPH animal models (Prasad et al., 2008 ▶; Mbaka et al., 2013 ▶).

SJ seed extract which is recognized for its potent anti-inflammatory and antioxidant properties, effectively ameliorated prostatic hyperplasia by causing marked degenerative changes in prostatic tissue stromal and epithelial cells of the extract-treated animals. The extract also exhibited significant anti-oxidant effect with considerable prophylactic activity. However, further investigation is needed for its validation.
